# Complete coding sequence of dengue virus serotype 4 isolated from field-caught mosquitoes in Thailand

**DOI:** 10.1590/0074-02760170022

**Published:** 2017-08

**Authors:** Thikhumporn Sittivicharpinyo, Passorn Wonnapinij, Wunrada Surat

**Affiliations:** 1 Kasetsart University, Faculty of Science, Department of Genetics, Evolutionary Genetics and Computational Biology Research Unit, Bangkok, Thailand; 2 Kasetsart University, National Research University-Kasetsart, Centre for Advanced Studies in Tropical Natural Resources, Bangkok, Thailand

**Keywords:** DENV-4, next generation sequencing, amino acid variation, field-caught mosquitoes and Thailand

## Abstract

This report is the first to characterise the complete coding sequence of a dengue virus serotype 4 (DENV-4) genotype I that was isolated from field-caught mosquitoes from an endemic area in Thailand in June 2013. The sequence was assembled from high-throughput sequencing reads generated by Illumina HiSeq. Three out of four observed intra-sample variants caused an amino acid variation in *C*, *NS2B*, and *NS5* genes. The C4279T variant located in the *NS2B* gene can indirectly affect the proteolytic activity of the NS3 protein. The sequence provided in this study might be useful for the epidemiological study of DENV-4.

Dengue virus (DENV) is an important arbovirus transmitted to humans by infected *Aedes* mosquitoes (WHO 2016). This virus has been classified into four different serotypes, namely DENV-1, 2, 3 and 4, and causes a wide range of diseases from non-specific febrile illness to severe dengue haemorrhagic fever (DHF) or shock syndrome (Bäck & Lundkvist 2013). These four serotypes co-circulate in Thailand (Klungthong et al. 2004) that is considered as one of the main reservoirs of DENV-4 worldwide (Nunes et al. 2012). DENV-4 has been further differentiated into four genotypes, namely I, II, III, and sylvatic (Klungthong et al. 2004), all of which have originated in Southeast Asia (Nunes et al. 2012). Every genotype, except the sylvatic, has been observed in Thailand (Klungthong et al. 2004). DENV-4, as a secondary infection, has been associated with DHF (Klungthong et al. 2004, Dewi et al. 2014). Although epidemiological and genomic sequence reports of DENV-4 are less than those of other DENV serotypes, complete sequences of genotypes I, II, III, and sylvatic isolated from human blood samples have been reported (Klungthong et al. 2004, Zhao et al. 2012, do Nascimento et al. 2016). To the best of our knowledge, the complete coding sequence of DENV-4 isolated from field-caught mosquitoes has not been reported until date.

In this study, DENV-4 (named CTI2-13) was isolated from field-caught mosquitoes collected from an endemic area in the Chanthaburi province, Thailand, in 2013. Total RNA was extracted from a pool of three mosquitoes using the RNeasy Mini Kit (Qiagen, Germany), and cDNA was generated by the SuperScript^®^ III First-Strand synthesis system (Invitrogen^™^, United States of America) according to the manufacturer’s instructions. Long-range polymerase chain reaction (PCR) products covering the entire DENV-4 genome were amplified using four primer pairs leading to overlapped amplicons with the forward and reverse primers of the first and the last pair of primers bound to 5′- and 3′-untranslated regions (UTRs) of the DENV-4 genome, respectively (Chin-inmanu et al. 2012). All four PCR products were diluted to the same concentration and pooled in equimolar amounts. Subsequently, paired-end libraries were prepared and sequenced by Illumina HiSeq.

A total of 652,427 clean paired-end reads with an average length of 150 nucleotides (nt) were obtained. The quality of the sequence reads was evaluated by FastQC (Andrews 2010). Low-quality score bases were excluded by Trimmomatic (Bolger et al. 2014) providing 450,628 qualified paired-end reads with an average length of 100-150 nt. The qualified reads were mapped to the NCBI DENV-4 reference sequence (NC_002640.1) using BWA (Li & Durbin 2009). Variant sites and a consensus sequence were generated by Samtools (Li & Durbin 2009, Li 2011). Numbers of non-synonymous nucleotide and amino acid (aa) differences were computed using MEGA7 (Kumar et al. 2016). The total of 189 complete coding sequences of DENV-4 genotype I, II, III and sylvatic from GenBank were included into the phylogenetic analysis using Neighbour-joining method (Saitou & Nei 1987). The bootstrap values were derived from 1000 replications.

The nearly complete DENV-4 genome sequence - composed of a partial 5′-UTR (99 nt), a partial 3′-UTR (353 nt), and the complete coding sequence (10164 nt) - was 10,616 nt in length. The average sequencing depth of the whole consensus sequence was 224x. The average sequencing depth of every protein-coding gene was above 200x, except for *NS2B* and *NS3* genes that had average sequencing depths of 164x and 59x, respectively. The average sequencing depth of the 5′- and 3′- UTRs was 171x and 207x, respectively. As shown in the Figure, phylogenetic analysis of the complete coding sequence suggests that the isolated DENV-4 belongs to genotype I. Our sample is closely related to DENV-4 genotype I strains previously isolated from human samples in Thailand, (AY318990, AY318992, KR922405), Cambodia, (KF955510, JN638570, JN638571, JN638572) and Brazil (JQ513345) (Saitou & Nei 1987, Klungthong et al. 2004, Nunes et al. 2012). However, our sample is not grouped into the same clade as these samples, suggesting that the mosquitoes collected in this study may carry a new sub-lineage of DENV-4.[Fig f01]

**Figure f01:**
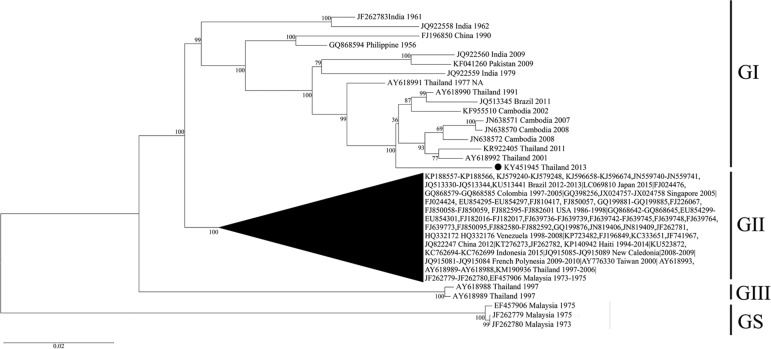
Neighbour-joining phylogenetic tree of the complete coding sequence of dengue virus serotype 4 (DENV-4). Our sample, named CTI2-13 (KY451945), is represented by a black dot. 189 complete DENV-4 coding sequences were retrieved from GenBank for the phylogenetic analysis. The numbers on the branches represent bootstrap supports calculated from 1,000 replicates. GI, GII, GIII, and GS represent genotype I, II, III, and sylvatic, respectively.

There were four positions in the protein-coding genes showing variation within the sample. These intra-sample variants were C403T, C4279T, G6125A/T, and G9992A located in *C*, *NS2B*, *NS3,* and *NS5* genes, respectively. Unlike the intra-host variation previously reported by do Nascimento et al. (2016), we observed no intra-sample variation in the *E* or *NS1* genes. This difference could be the result of different genotypes, sample types, or criteria for variant calling. The position of variants presented here was based on the NCBI reference sequence (NC_002640.1). As shown in the Table, the minor allele frequencies of these variants were at least 30%. Every variant, except the G6125A/T variant, generated two types of aas. Interestingly, the V49A variation generated by the C4279T variant is located in the NS2B protein within the region that interacts with the protease domain of the NS3 protein (Sessions et al. 2015). Thus, this aa change would possibly affect the proteolytic activity of the NS3 protein. We also observed an intra-sample variation in the *NS3* gene (the G6125A/T) located in the protease domain-encoding region (Natarajan 2010). However, this variant did not cause an aa change. This intra-sample genetic variation could be generated by either an error-prone replication of DENV within a mosquito vector (Sessions et al. 2015, Sim et al. 2015) or a co-circulation of multiple forms of DENV-4 in the area (Zhao et al. 2010). Nevertheless, the limitations of our study design do not allow to address this question. Although we could not identify whether this intra-sample variation represented an intra-host variation, DENV genetic variations may increase the incidence of dengue disease in the area. The variants reported herein might not affect the incidence of dengue disease if they are lost in the DENV population bottleneck produced during abdomen-to-salivary gland or mosquito-to-human transmission (Sim et al. 2015).

**TABLE:** Intra-sample genetic variation presented in the dengue virus serotype 4 (DENV-4) genotype I isolated from the pooled field-caught mosquitoes named CTI2-13

Site^*a*^	Gene	Cov^*b*^	Maj^*c*^	Min^*d*^	Min freq^*e*^	Type	Pos^*f*^	aa^*g*^
403	*C*	236	t	c	0.50	NS	2	I101T
4279	*NS2B*	245	t	c	0.46	NS	2	V49A
6125	*NS3*	46	a	t	0.39	S	3	V534V
9992	*NS5*	289	a	g	0.33	NS	3	I810M

a: nucleotide position that was based on the NCBI reference sequence (NC_002640.1); b: coverage (COV); c: major allele (maj); d: minor allele (min); e: minor allele frequency (min freq); f: position in the codon (pos); g: amino acid (aa) position that was based on position in the protein product of the gene; NS: nonsynonymous substitution; S: synonymous substitution.

In summary, this is the first report on the complete coding sequence of DENV-4 genotype I isolated from field-caught mosquitoes in Thailand. Several intra-sample variants were observed, at least one of them potentially affecting the activity of the NS3 protein of the virus. Our results support the use of high-throughput sequencing to detect intra-sample genetic variation, which might help gain insight into the epidemiology of diseases caused by viral infection.

The complete coding sequence, as well as the partial 5′- and 3′- UTRs, of DENV-4 genotype I (named CTI2-13) isolated from the field-caught mosquitoes in Thailand was submitted to GenBank, under the accession number KY451945.
